# Draft Genome Sequence of *Mycobacterium massiliense* Strain M159, Showing Phenotypic Resistance to β-Lactam and Tetracycline Antibiotics

**DOI:** 10.1128/genomeA.00669-13

**Published:** 2013-08-29

**Authors:** Yun Fong Ngeow, Mee Lian Leong, Yan Ling Wong, Guat Jah Wong, Joon Liang Tan, Wei Yee Wee, Chia Sui Ong, Yong Kek Pang, Siew Woh Choo

**Affiliations:** Department of Medical Microbiology, Faculty of Medicine, University of Malaya, Kuala Lumpur, Malaysiaa; Dental Research and Training Unit, Faculty of Dentistry, University of Malaya, Kuala Lumpur, Malaysiab; Genome Informatics Research Laboratory, High Impact Research (HIR) Building, University of Malaya, Kuala Lumpur, Malaysiac; Faculty of Information Science and Technology, Multimedia University, Melaka, Malaysiad; Department of Medicine, Faculty of Medicine, University of Malaya, Kuala Lumpur, Malaysiae

## Abstract

*Mycobacterium massiliense* is a nontuberculous mycobacterium associated with human infections. We report here the draft genome sequence of *M. massiliense* strain M159, isolated from the bronchial aspirate of a patient who had a pulmonary infection. This strain showed genotypic and *in vitro* resistance to a number of tetracyclines and beta-lactam antibiotics.

## GENOME ANNOUNCEMENT

The rapidly growing *Mycobacterium* species *M. abscessus* is an established human pathogen (1). Three subspecies, *M. abscessus* *sensu stricto*, *M. massiliense*, and *M. bolletii*, have been recognized for many years (2, 3, 4), but recently, in 2011, Leao and colleagues (5) proposed a reclassification into two subspecies, *M. abscessus* subspecies *abscessus* and *M. abscessus* subspecies *bollettii*, which comprises the former *M. bolletii* and *M. massiliense*. There are, however, distinct phenotypic features that distinguish *M. bolletii* from *M. massiliense* as well as DNA sequences that differentiate these two former subspecies in phylogenetic studies (6, 7). Here, we report the draft genome sequence of M159, a strain of *M. abscessus* subspecies *bolletii* referred to here as *M. massiliense* for ease of discussion.

Strain M159 was isolated from the bronchial aspirate of a 75-year-old male chronic smoker with prostatic carcinoma. He presented with a cough and chest radiograph signs of lung infection, which improved after a 2-week course of ciprofloxacin. The isolate was identified as *Mycobacterium massiliense* by its rapid growth (within 5 days in the mycobacterial growth indicator tube [MGIT]_960_ liquid culture system), acid-fast staining, and clustering with *M. massiliense* reference strains in *rpoB*- and *hsp65*-based phylogenetic analyses. MIC determinations using the Sensititre RGMyco plate (TREK Diagnostic Systems, Cleveland, OH) showed *in vitro* resistance to doxycycline, minocycline, ceftriaxone, cefepime, and amoxicillin-clavulanic acid and intermediate resistance to cefoxitin and imipenem but susceptibility to ciprofloxacin, clarithromycin, amikacin, and linezolid.

The genome sequence of M159 was determined using the Illumina GA IIx platform. The 20,229,857 raw read sequences generated were processed and assembled using Genomics Workbench 4.9. This resulted in 118 contigs with an *N*_50_ value of 115,707 bp and a draft genome sequence of 4,935,864 bp with a G+C content of 64%. Functional annotation using the Rapid Annotations using Subsystems Technology (RAST) Server (8) predicted 4,864 coding sequences (CDS) and 51 RNAs (48 tRNAs and 3 rRNAs). Of the 1,540 (32%) functional genes assigned to functional categories, most are involved in amino acids and derivatives (427 genes), followed by cofactors, vitamins, and prosthetic groups (347 genes), carbohydrates (284 genes), and fatty acids, lipids, and isoprenoids (256 genes). In contrast, there was only 1 putative gene for motility and chemotaxis, and there were 49 genes associated with virulence, disease, and defense. In agreement with its antibiotic susceptibility pattern, M159 showed the typical truncated *erm*(41) gene associated with susceptibility to macrolides (9), *bla* genes for resistance to cephalosporins and clavulanic acid inhibition, and three genes for tetracycline resistance, namely, the *tetA* and *tetC* genes encoding efflux proteins and an intact *tetR* gene, which is associated with the upregulation of the expression of efflux proteins in the presence of tetracycline (10).

### Nucleotide sequence accession numbers. 

The *M. massiliense* strain M159 genome sequence has been deposited in NCBI GenBank/DDBJ/EMBL under the accession number AJSD00000000. The version described in this paper is the first version, under accession number AJSD01000000.

## References

[1] Medjahed H Gaillard JL Reyrat JM 2010 *Mycobacterium abscessus*: a new player in the mycobacterial field. Trends Microbiol. 18:117–123. 10.1016/j.tim.2009.12.007 20060723

[2] Adékambi T Drancourt M 2004 Dissection of phylogenetic relationships among 19 rapidly growing *Mycobacterium* species by *16S rRNA, hsp65, sodA, recA* and *rpoB* gene sequencing. Int. J. Syst. Evol. Microbiol. 54:2095–2105 15545441 10.1099/ijs.0.63094-0

[3] Adékambi T Reynaud-Gaubert M Greub G Gevaudan MJ La Scola B Raoult D Drancourt M 2004 Amoebal coculture of “*Mycobacterium massiliense*” sp. nov. from the sputum of a patient with hemoptoic pneumonia. J. Clin. Microbiol. 42:5493–5501. 10.1128/JCM.42.12.5493-5501.2004 15583272 PMC535245

[4] Adékambi T Berger P Raoult D Drancourt M 2006 rpoB gene sequence-based characterization of emerging non-tuberculous mycobacteria with descriptions of *Mycobacterium bolletii* sp. nov., *Mycobacterium phocaicum* sp. nov. and *Mycobacterium aubagnense* sp. nov. Int. J. Syst. Evol. Microbiol. 56:133–143. 10.1099/ijs.0.63969-0 16403878

[5] Leao SC Tortoli E Euzéby JP Garcia MJ 2011 Proposal that *Mycobacterium massiliense* and *Mycobacterium bolletii* be united and reclassified as *Mycobacterium abscessus* subsp. *bolletii* comb. nov., designation of *Mycobacterium abscessus* subsp. *abscessus* subsp. nov. and emended description of *Mycobacterium abscessus*. Int. J. Syst. Evol. Microbiol. 61:2311–2313. 10.1099/ijs.0.023770-0 21037035

[6] Macheras E Roux AL Bastian S Leão SC Palaci M Sivadon-Tardy V Gutierrez C Richter E Rüsch-Gerdes S Pfyffer G Bodmer T Cambau E Gaillard JL Heym B 2011 Multilocus sequence analysis and rpoB sequencing of *Mycobacterium abscessus* (sensu lato) strains. J. Clin. Microbiol. 49:491–499. 10.1128/JCM.01274-10 21106786 PMC3043527

[7] Kim HY Kim BJ Kook Y Yun YJ Shin JH Kim BJ Kook YH 2010 *Mycobacterium massiliense* is differentiated from *Mycobacterium abscessus* and *Mycobacterium bolletii* by erythromycin ribosome methyltransferase gene (*erm*) and clarithromycin susceptibility patterns. Microbiol. Immunol. 54:347–353. 10.1111/j.1348-0421.2010.00221.x 20536733

[8] Aziz RK Bartels D Best AA DeJongh M Disz T Edwards RA Formsma K Gerdes S Glass EM Kubal M Meyer F Olsen GJ Olson R Osterman AL Overbeek RA McNeil LK Paarmann D Paczian T Parrello B Pusch GD Reich C Stevens R Vassieva O Vonstein V Wilke A Zagnitko O 2008 The RAST server: rapid annotations using subsystems technology. BMC Genomics 9:75. 10.1186/1471-2164-9-75 18261238 PMC2265698

[9] Nash KA Brown-Elliott BA Wallace RJ Jr. 2009 A novel gene, *erm*(41), confers inducible macrolide resistance to clinical isolates of *Mycobacterium abscessus* but is absent from *Mycobacterium chelonae*. Antimicrob. Agents Chemother. 53:1367–1376. 10.1128/AAC.01275-08 19171799 PMC2663066

[10] Beck CF Mutzel R Barbé J Müller W 1982 A multifunctional gene (*tetR*) controls tn10-encoded tetracycline resistance. J. Bacteriol. 150:633–642 6279565 10.1128/jb.150.2.633-642.1982 PMC216410

